# Trans‐Cervical Ultrasound Imaging of the Adult Palatine Tonsils

**DOI:** 10.1002/jmrs.70139

**Published:** 2026-10-22

**Authors:** Michelle Fenech, Tristan Reddan

**Affiliations:** ^1^ College of Clinical Sciences, Health, Medical and Applied Sciences Central Queensland University Brisbane Queensland Australia; ^2^ Medical Imaging and Nuclear Medicine, Queensland Children's Hospital Children's Health Queensland Hospital and Health Service South Brisbane Queensland Australia; ^3^ School of Clinical Sciences, Faculty of Health Queensland University of Technology Brisbane Queensland Australia

**Keywords:** intratonsillar abscess, peritonsillar abscess, peritonsillar cellulitis, quinsy, superior constrictor muscle, ultrasonography

## Abstract

Trans‐cervical ultrasound of the palatine tonsils provides a non‐invasive technique to image the adult tonsils and the associated peritonsilar space to investigate intratonsillar and peritonsillar abscesses. The peritonsilar space, where peritonsillar cellulitis and abscesses are located is readily identified sonographically between the palatine tonsil and the superior constrictor muscle, which can be used as a helpful sonographic landmark. An understanding of the sonographic anatomy, technique, landmarks and imaging appearances is essential to correctly perform and interpret ultrasound imaging of the adult palatine tonsils which is unpacked in this narrative review.

## Graphical abstract

Ultrasound imaging of the adult palatine tonsils using a trans‐cervical approach can be used to assess the tonsillar region for intratonsillar and peritonsillar abscesses. An understanding of the sonographic anatomy, technique and tonsillar pathology is required to perform and interpret imaging and inform patient management.

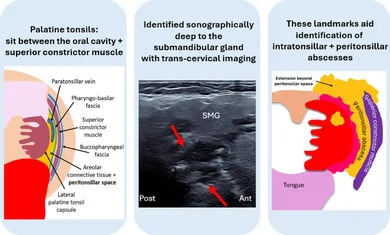

## 1 Introduction

The tonsils are located at the entrance to the upper respiratory and gastrointestinal tracts, and as part of the immune system act as a first line of defence against ingested or inhaled pathogens [1, 2]. Tonsillitis is usually a self‐limiting condition, and diagnosed clinically [2]. However, tonsillitis can progress to intra‐tonsillar and peritonsillar abscess [3]. Known as a quinsy, peritonsillar abscesses can progress unfavourably if untreated; they can lead to aspiration pneumonia, extend into deep neck spaces and the mediastinum, cause jugular vein thrombophlebitis and cavernous sinus thrombosis and acute airway obstruction [2, 4, 5]. Although physical examination is used to diagnose tonsillitis, clinically distinguishing between peritonsillar cellulitis, intratonsillar and peritonsillar abscess can be challenging [6]. A timely and accurate differentiation between peritonsillar cellulitis and abscess formation is needed to guide appropriate management which includes antimicrobial treatment and possibly abscess drainage [3, 5, 7].

Computed tomography (CT) can be used to define the extent of extra tonsillar infection spread, particularly when airway compromise is suspected, or there is clinical deterioration following treatment [5, 6, 8, 9, 10, 11]. Despite its accuracy, CT uses ionising radiation, requires intravenous contrast which can be associated with adverse reactions, and cannot be used at the patient bedside [5]. With CT imaging, it can also be difficult to distinguish between the tonsils and surrounding tissues including the anterior and posterior tonsillar pillars, and structures that form the boundaries of the peritonsillar space [12, 13]. Magnetic resonance imaging (MRI) is generally not readily available in the emergency setting and encounters artefacts from breathing and pulsations of nearby vessels, which can make distinguishing the tonsils, peritonsillar space and surrounding structures difficult [5, 12].

In contrast trans‐cervical ultrasound of the palatine tonsils offers advantages as a non‐invasive imaging technique, able to delineate the tonsils and peritonsillar tissues, at the patient bedside [5]. Patients tolerate trans‐cervical ultrasound imaging well, and its use is established in paediatric populations to image the tonsillar and surrounding tissues [4, 6, 7, 9, 14, 15]. Intra‐oral sonography of the palatine tonsils, where the ultrasound transducer is placed within the mouth, is not as well tolerated by patients, particularly those that cannot open their mouth fully, due to inflammation and pain (trismus), a common symptom of tonsillitis, cellulitis, intratonsillar and peritonsillar abscess [16].

Tonsillar ultrasound has been adopted by non‐radiologic health professionals to investigate the risk of obstructive sleep apnoea due to tonsillar hypertrophic enlargement [17]. However, there is a paucity of literature outlining the use of trans‐cervical ultrasound imaging of adult tonsils for the diagnosis of tonsillar and peritonsillar pathology. As ultrasound guidance of drainage of tonsillar or peritonsillar abscesses can also be used as a treatment regime, an improved understanding of the technique of sonographically imaging the palatine tonsils and surrounding tissues is required [6, 11, 16].

To undertake a tonsillar sonographic assessment, a good understanding of the sonographic anatomy, imaging technique and sonographic appearances of the palatine tonsils and the peritonsillar tissues is required. This narrative review paper will unpack the sonographic technique to image the adult palatine tonsils, tonsillar bed, and peritonsillar space, using a trans‐cervical technique. Additionally, it will highlight important structures which can be used as sonographic landmarks to identify the peritonsillar space, where peritonsillar abscesses form. Ethics approval was not required for this narrative review, as it did not involve human participants or any patient data.

## 2 Anatomy

### 2.1 Anatomy of the Tonsils Within the Pharynx

There are four tonsils which include the palatine, pharyngeal, lingual and tubal tonsils [18]. Collectively, they form a ringed arrangement of lymphoid tissue known as Waldeyer's lymphatic (tonsillar) ring; it spans the nasopharynx and the oropharynx, including the base of tongue and soft palate [1, 18, 19]. Distinguishing between the tonsils is important when imaging them. The palatine tonsils are differentiated from other tonsils due to their relative anatomical position in the pharynx [18]. Extending from the skull base to the inferior border of the cricoid cartilage, the pharynx is divided into three main regions: the nasopharynx, oropharynx and hypopharynx [10, 20].

The nasopharynx extends from the base of the skull to the level of the soft palate, posterior to the nasal cavity, and houses the unpaired pharyngeal tonsil and paired tubal tonsils [10, 18, 20]. The oropharynx (mid‐pharynx) extends inferiorly from the junction of the soft and hard palates to the level of the hyoid bone and epiglottis, and contains the unpaired lingual and paired palatine tonsils [20] (Figure 1).

**FIGURE 1:**
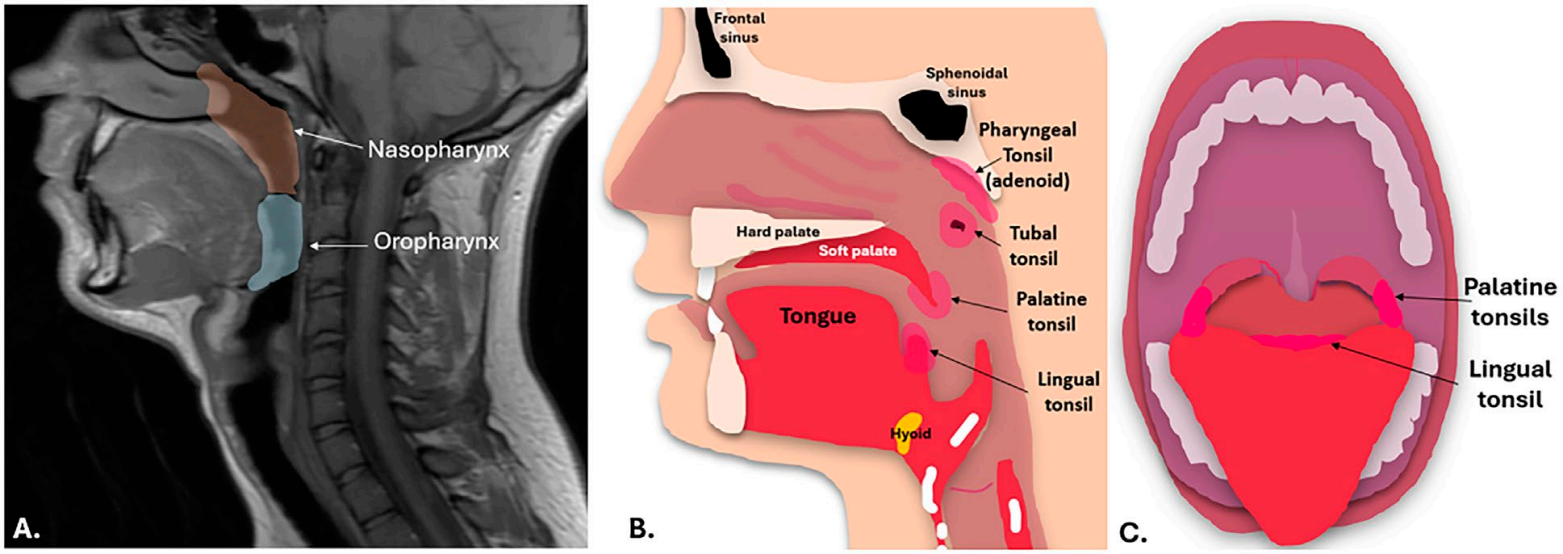
Relative positions of the tonsils and nasopharynx and oropharynx. (A) Midsagittal magnetic resonance (MR) image indicating the position of the nasopharynx and oropharynx. (B) Sagittal diagram of the oropharynx and nasopharynx regions indicating the positions of all tonsils. (C) Figure of the open mouth showing the positions of the palatine and lingual tonsils.

#### 2.1.1 Pharyngeal Tonsil (Adenoid)

The unpaired pharyngeal or nasopharyngeal tonsil is typically called the adenoid [19]. It is a single mass of lymphoid tissue centrally located at the junction of the roof and posterior wall of the nasopharynx [18, 19]. It is located postero‐superior to the palatine tonsils and the nasal apertures (choanae) [18, 19]. Adenoids reach a maximum size in children aged 3 to 7 years, after which they usually undergo appreciable atrophy [21]. Paediatric adenoid enlargement can occur as an abnormal immune response; it can lead to chronic inflammation which can spread to adjacent mucosal areas including the pharyngo‐basilar fascia and cause obstruction of the nasopharynx and the opening of the eustachian tubes [19, 21]. This can lead to obstructive sleep apnoea, chronic sinusitis, otitis media with effusion, malocclusions, craniofacial developmental anomalies, and disorders associated with physical and intellectual development [19].

#### 2.1.2 Tubal Tonsils

The paired tubal tonsils, also called lateral cords, Gerlach or Eustachian tonsils, are located in the lateral wall of the nasopharynx, immediately posterior to the Eustachian tube orifice (connecting the middle ear to the nasopharynx) [18]. They form the lateral aspect of Waldeyer's ring and sit superior to the palatine tonsils [18].

#### 2.1.3 Lingual Tonsil

The unpaired lingual tonsil refers to midline lymphoid nodules located within the submucosa of the posterior third of the tongue base, between the epiglottis and the tonsillar pillars [22, 23]. The lingual tonsil sits medial to the palatine tonsils. Forming the inferior part of Waldeyer's ring, the lingual tonsil is responsible for the irregular appearance of the posterior tongue surface [22]. Lingual tonsillar hypertrophy is rare but has been associated with an elevated body mass index and gastro‐oesophageal reflux [23].

#### 2.1.4 Palatine Tonsils

The paired palatine tonsils are commonly referred to as ‘the tonsils’, or faucial tonsils due to their location in the tonsillar fossa, also called the fauces [9]. They are oval or walnut shaped, solid, lymphoid glands located bilaterally in the lateral walls of the oropharynx; forming the lateral part of Waldeyer's ring [2, 18]. The tonsillar fauces are located laterally between the soft palate and the base of the tongue [18]. They are bordered anteriorly and posteriorly by mucosal folds, commonly referred to as tonsillar pillars [9, 18]. The tonsillar pillars are formed by the palatoglossal (anterior) and palatopharyngeal (posterior) muscles, also referred to as the anterior palatoglossal and the posterior palatopharyngeal arches [2] (Figure 2).

**FIGURE 2:**
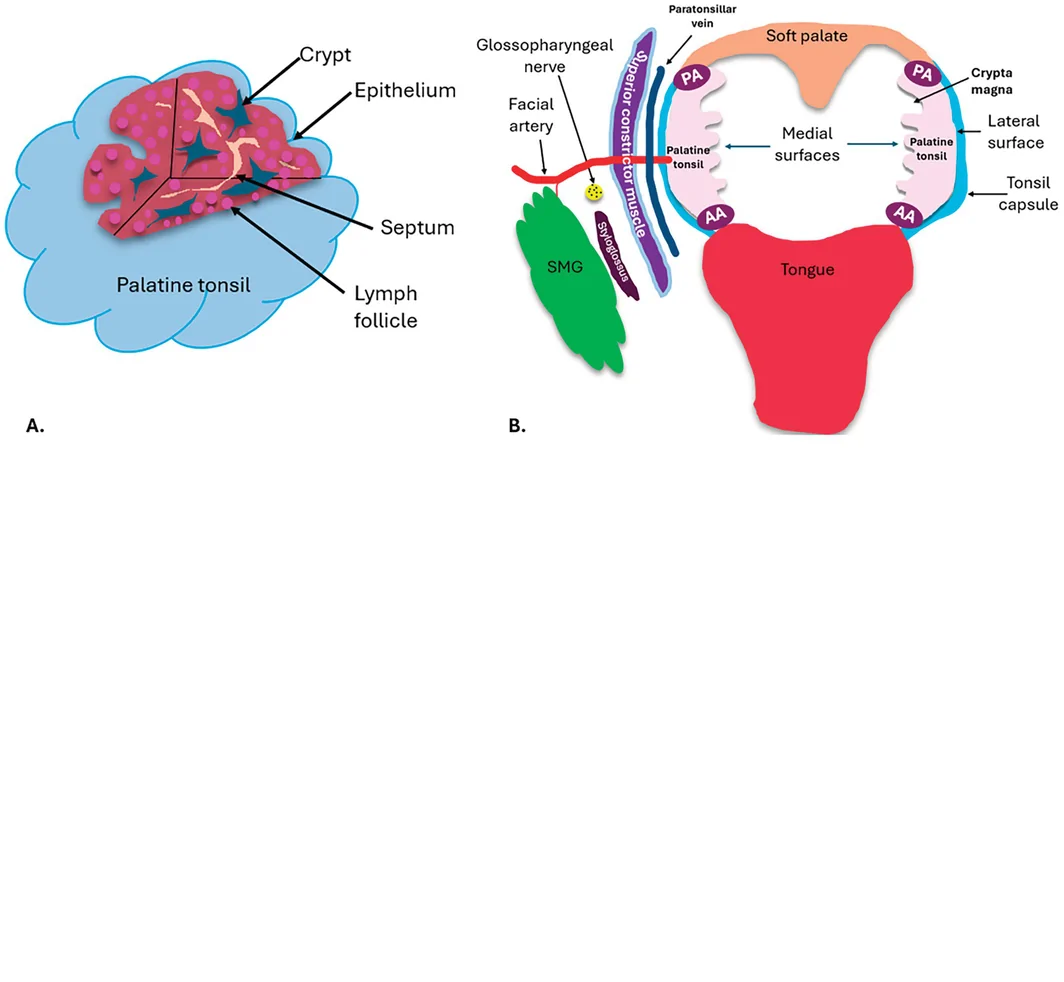
The palatine tonsils. (A) The palatine tonsil has a lobulated outline and a folded structure of solid parenchyma and crypts (recesses). (B) The relative position of the palatine tonsil in the fauces, between the anterior arch (AA) which is the anterior palatoglossus muscle, and the posterior arch (PA), which is the posterior palatopharyngeal muscle; these muscles form respective pillars (arches). The palatine tonsils have medial and lateral surfaces. The crypta magna is the largest crypt (fold) on the medial surface of the palatine tonsil. (C) Longitudinal view of the medial border of the palatine tonsil between the anterior and posterior arches. (D) The lateral margin of the palatine tonsil is bordered by the tonsillar capsule, and lateral to this sits the peritonsillar space, which contains the paratonsillar vein, and this is bordered more laterally by the superior constrictor muscle. SMG, submandibular gland.

The palatine tonsils are rich in lymphocytes, with the tonsil surface covered by epithelium [1]. Deep to the epithelium, the tonsillar tissue is organised into lymphoid follicles with germinal centres [18]. The palatine tonsils have two surfaces, important to appreciate with imaging: medial and lateral [18]. The free medial surface of the palatine tonsil projects into the oropharynx [9].

The tonsillar epithelium dips into the tonsil to form crypts, which form irregular, numerous, blind‐ended pits or crevices, which increase the tonsillar surface area and on the medial surface can trap microorganisms, bacteria, viruses and foreign material such as food debris entering the oropharynx [4]. The crypts give the tonsil a lobulated appearance on their medial free edge [18]. There are approximately 8–20 crypts which have primary and secondary branches which extend through the tonsil [3]. The largest crypt, called the crypta magna (intratonsillar cleft), is located on the supero‐medial aspect of the tonsil [18]. If material gets impacted within crypts, it can lead to the development of tonsilloliths and/or acute and recurring tonsillitis [3]. Sonographically, the crypts contribute to the palatine tonsil having a striated appearance.

The lateral surface of the palatine tonsil is covered by a fibrous capsule [24]. Lateral to this tonsillar capsule lies the important peritonsillar tissues which contain loose areolar connective tissue, the paratonsillar vein, pharyngo‐basilar fascia, the superior constrictor muscle and most laterally the buccopharyngeal fascia [4, 18, 25]. The loose areolar tissue allows for movement of the tonsils and makes tonsillectomy procedures possible [18]. Sonographically, healthy palatine tonsils are virtually inseparable from the adjacent loose areolar tissue [24].

Healthy palatine tonsils, free of inflammation and infection, are clinically observed to have a pink colour [2]. They are well vascularised and the tonsillar and peritonsillar vasculature should be assessed sonographically with colour Doppler; however, it is not well described in imaging literature. The main arterial supply is provided via the tonsillar branch of the facial artery (which arises from the external carotid artery) [26]. This tonsillar branch ascends between the styloglossus and medial pterygoid muscles to pierce the superior constrictor muscle to supply the palatine tonsil [26].

Further blood supply of the palatine tonsil is derived from the dorsal lingual branch of the lingual artery, which supplies its inferior and posterior aspects [27]. It courses between the hyoglossus muscle and the middle constrictor muscle to pass around the root of the tongue to reach the tonsil [18]. Palatine tonsillar venous drainage occurs via the paratonsillar vein, which drains into the facial vein [28].

The palatine tonsils are innervated by the glossopharyngeal and maxillary (2nd division of trigeminal nerve) nerves [18]. These nerves can be identified sonographically, may get irritated by nearby infection, and are often underappreciated. The glossopharyngeal nerve passes lateral to the superior constrictor muscle [28]. Because the glossopharyngeal nerve also provides sensation to the middle ear (via the tympanic branch), patients with tonsillitis or recent tonsillar surgery often complain of otalgia (ear pain) [29]. The glossopharyngeal nerve also gives off a lingual branch, which enters the tongue base and supplies taste sensation to the posterior third of the tongue [29]. The lingual branch runs close to the tonsillar fossa and is susceptible to injury with tonsillectomy [18].

Lymphadenopathy can be clinically confused for peritonsillar abscess, particularly when unilateral [30]. Ultrasound imaging can be helpful in differentiating lymphadenopathy from peritonsillar abscess [30]. Unlike other lymphoid structures such as lymph nodes, the palatine tonsils do not have an afferent lymphatic network [18]. Palatine tonsillar lymph drains into the jugulodigastric and retropharyngeal lymph nodes, which can become reactively enlarged with tonsillitis and observed with medical imaging [31].

### 2.2 Anatomy of Peritonsillar Tissues

#### 2.2.1 The Superior Constrictor Muscle

The superior constrictor muscle is an important landmark for imaging the palatine tonsils and peritonsillar space. It is the uppermost and thinnest of three circular muscles (superior, middle and inferior constrictor muscles) forming the muscular wall of the pharynx [20]. A thin sheet‐like muscle, it forms the posterior and lateral walls of the oropharynx [27]. Originating from the medial mandible and pterygoid bone, the superior constrictor muscle wraps around the pharynx postero‐laterally, inserting onto the median pharyngeal raphe at the posterior pharynx [20]. The main function of the constrictor muscles is to facilitate deglutition, as they constrict the pharyngeal wall during swallowing [20]. Sonographically, the superior constrictor muscle forms the postero‐lateral wall of the peritonsillar space around the palatine tonsil (Figure 3).

**FIGURE 3:**
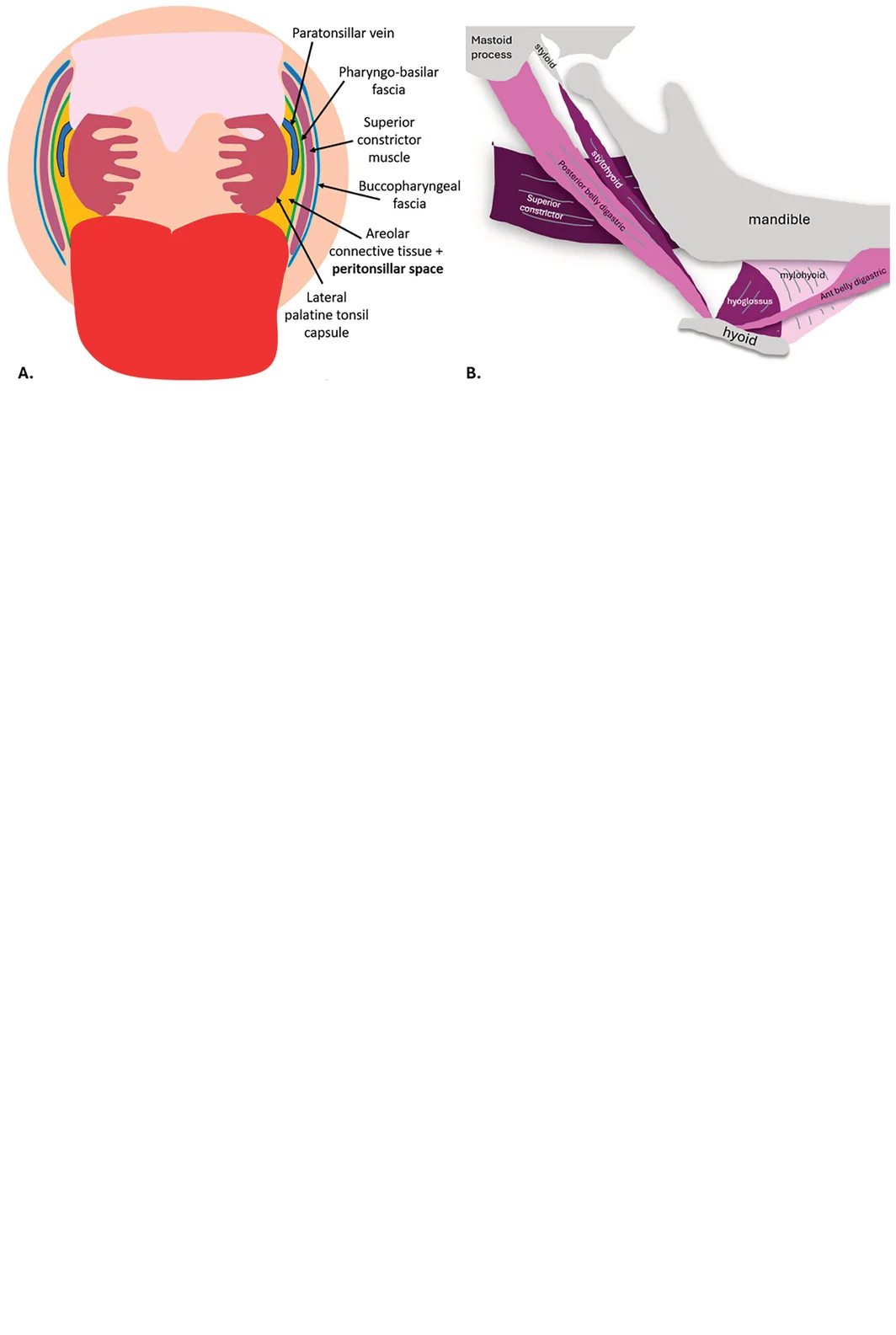
The superior constrictor muscle as a sonographic landmark for imaging the palatine tonsil. (A) Position of the palatine tonsils and lateral structures including the lateral tonsil capsule, superior constrictor muscle, region of the areolar loose connective tissue and the peritonsillar space. (B) The superior constrictor muscle is located medial to the mandible, and relative to other muscles used as sonographic landmarks to image the submandibular salivary gland (SMG). (C) Transducer positioning for a trans‐cervical long axis imaging approach of the palatine tonsil. (D) A long axis image of the SMG and deeper located palatine tonsil, outlining the position of the superior constrictor muscle. (E) Discrimination between the peritonsillar and parapharyngeal spaces in an axial view through the neck. ANT, anterior aspect of image; ICA, internal carotid artery; IJV, internal jugular vein; POST, posterior aspect of image; SLG, sublingual salivary gland; SP, styloid process.

#### 2.2.2 The Peritonsillar Space

The peritonsillar space sits between the palatine tonsil capsule and the superior constrictor muscle; and importantly, is where peritonsillar abscesses form [4, 10, 14]. Anteriorly it is bordered by the palatoglossus muscle/anterior tonsillar pillar/palatoglossal arch [3]. Posteriorly, it is bordered by the palatopharyngeus muscle/posterior tonsillar pillar/palatopharyngeal arch [30]. Superiorly, the peritonsillar space extends to the level of the hard palate, and inferiorly to the piriform sinus (lateral channel for the passage of food during swallowing) [32].

#### 2.2.3 Discrimination Between the Peritonsillar and Parapharyngeal Spaces

The peritonsillar space must not be confused with the parapharyngeal space which is a deep potential space between the base of the skull and hyoid bone serving as a pathway for the spread of infections and tumours [33, 34]. The parapharyngeal space contains fat and neurovascular structures and is located lateral to the peritonsillar space [35]. At the level of the palatine tonsil, the parapharyngeal space extends laterally from buccopharyngeal fascia, which lines the lateral aspect of the superior constrictor muscle; it is a critical imaging landmark for diagnosing lesions and infections [33, 35].

## 3 Trans‐Cervical Sonographic Technique

Trans‐cervical ultrasound describes the process of imaging the palatine tonsils from a submandibular approach, where the submandibular gland (SMG) is used as an acoustic window [15]. It is easier, more comfortable, and faster to perform compared to the alternative intra‐oral ultrasound imaging [9]. Intra‐oral imaging requires the ultrasound transducer to be covered with gel and a sterile cover and to be placed in the mouth. It can be difficult to ensure transducer coupling to the tonsil to ensure elimination of any air‐gap sonographic imaging artefacts. In some cases, the patient may be required to fill their mouth with water to produce an intra‐oral sonographic image, resulting in this approach being even less tolerated [30].

For trans‐cervical tonsillar imaging, the patient is positioned supine with their neck and chin extended [15]. The head is turned to the opposite side allowing transducer access under the chin to image the tonsil [15]. A high frequency linear transducer (> 12 MHz) is required to define the tonsillar tissue, its borders, and have sufficient penetration to image the surrounding peritonsillar tissues [36]. The tonsils are examined in both long and short axis planes, described relative to the axis of the mandible and SMG [36]. The palatine tonsils are displayed deep to the SMG, mylohyoid, and hyoglossus muscles, all important imaging landmarks to signpost the tonsil position [36].

### 3.1 Sonographic Appearance of the Healthy Adult Palatine Tonsil

Sonographically, the palatine tonsil appears rounded or ellipsoid, with subtly lobulated margins, and hypoechoic relative to the superficially located SMG [37]. Intermingling hypoechoic and hyperechoic linear bands may be seen resulting in a ‘trigoid’, ‘leopard print’ or striated appearance, due to the crypts, generally more obvious in children and younger adults [4, 9, 36]. In older adults, the palatine tonsil can be less obvious to distinguish from the oral cavity, which can make it challenging to discriminate and identify if its appearance is not appreciated. The tonsil can also be described sonographically as appearing like a ‘dirty bag of shadows’ (Figure 4).

**FIGURE 4:**
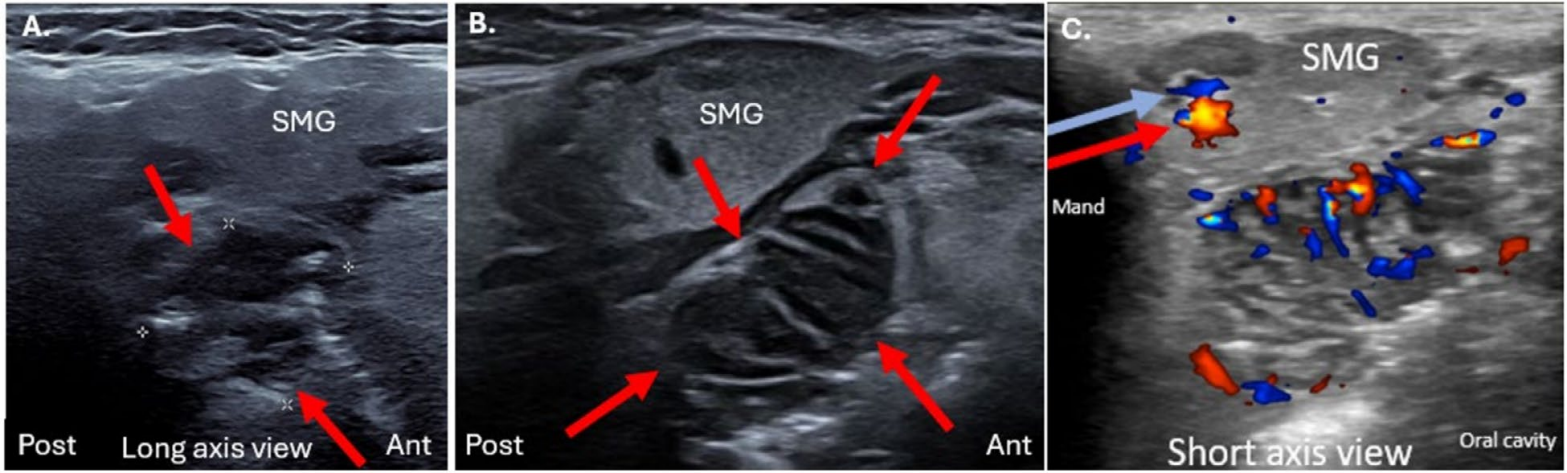
Trans‐cervical sonographic images of the palatine tonsil. (A) Long axis image of the palatine tonsil. The palatine tonsil (red arrows) is hypoechoic relative to the submandibular gland (SMG). It can be mistaken for part of the oral cavity, if its anatomy and sonographic appearance is not understood. (B) The striated appearance of a palatine tonsil (outlined by red arrows) in a younger adult in a long axis image. (C) Colour Doppler short axis image of the palatine tonsil, demonstrating internal vascularity of the tonsil and the facial artery (red arrow) and facial vein (blue arrow) along the lateral aspect of the SMG, superficial to the palatine tonsil when imaged from a trans‐cervical approach. Ant, anterior aspect of image; Mand, mandible; Post, posterior aspect of image; SMG, submandibular gland.

### 3.2 Sonographic Appearance of the Palatine Tonsil Using Colour Doppler

Colour and power Doppler are used to demonstrate normal baseline tonsillar and peritonsillar vascularity and distinguish nearby vascular structures [9, 36]. Internal vascularity of the palatine tonsil should be identified and comparison of palatine tonsillar vascularity between contralateral sides can be used and symmetry in vascularity noted when normal [9]. When an abscess is present (intra‐tonsillar or peri‐tonsillar), no internal abscess vascularity will be identified with colour Doppler; however, vascularity around an abscess border may be seen [5].

### 3.3 Sonographic Size of Palatine Tonsils

Healthy adult palatine tonsils sonographically appear smaller in size when compared to the nearby SMG; if they appear larger than the SMG, they can be qualitatively reported as enlarged. Sonographic measurements of the palatine tonsils can be acquired from long and short axis static images [15]. Applying callipers to the outer border of the tonsils can help to define their extent. The normal adult palatine tonsils are approximately 10–20 mm in diameter, with 20 mm typically measured in their longest dimension [4, 36]. There are, however, a paucity of studies reporting the sonographic size and volume of palatine tonsils, and further research to define the palatine tonsillar size sonographically is required.

Healthy palatine tonsil size should not differ between the right and left sides [38]. The palatine tonsils are usually more sonographically obvious in children [2]. They increase significantly in size from birth to 3 years of age [38]. From 3 to 12 years of age, the tonsils do not significantly increase in size [4, 38]. The volume of palatine tonsils in children can be correlated somewhat with weight and BMI [36]. Good correlation between sonographic volume measurements of paediatric tonsils (pre‐surgically) and volumetric measures of the same tonsils, once surgically removed, has been demonstrated [15]. Between 3 and 17 years of age, the palatine tonsil volume averages 2–3 cc [9].

### 3.4 Trans‐Cervical Sonographic Appearance of the Peritonsillar Space

The peritonsillar space is discriminated sonographically, using the superior constrictor muscle; this helps to define the lateral margin of the palatine tonsil and the peritonsillar space. The facial artery and vein, coursing along the lateral aspect of the SMG, may be demonstrated with colour Doppler, superficial to the tonsil [4]. Both sides of the neck should be examined to provide a contralateral comparison [4]. If difficulty is encountered sonographically localising the palatine tonsils, where, for example, the SMGs are not easily visualised, the internal jugular vein (IJV) and internal and external carotid arteries (ICA and ECA) can be used as alternate sonographic landmarks. They should be identified sonographically in the lower neck and tracked superiorly. Occasionally, the transducer may need to be angled inferiorly from the SMG to observe the palatine tonsil.

### 3.5 Long Axis Trans‐Cervical Sonographic Imaging of the Palatine Tonsil

To obtain long axis trans‐cervical imaging of the palatine tonsil, the transducer is placed under the chin, medial to the infero‐lateral aspect of the body of the mandible and angled superiorly in a sagittal oblique plane [15]. The posterior aspect of the transducer is aligned with the angle of the mandible [39]. Muscular landmarks to identify the tonsil include the mylohyoid and hyoglossus muscles; they sit anterior and deep to the SMG and anterior and superficial to the tonsil. Additionally, the posterior belly of the digastric muscle will be identified posterior to the SMG and superficial to the tonsil. The superior constrictor muscle should be identified posterolateral to the palatine tonsil. Depending on the angulation of the transducer, the SMG can also be identified superficial and anterior to the palatine tonsil [40] (Figure 5).

**FIGURE 5:**
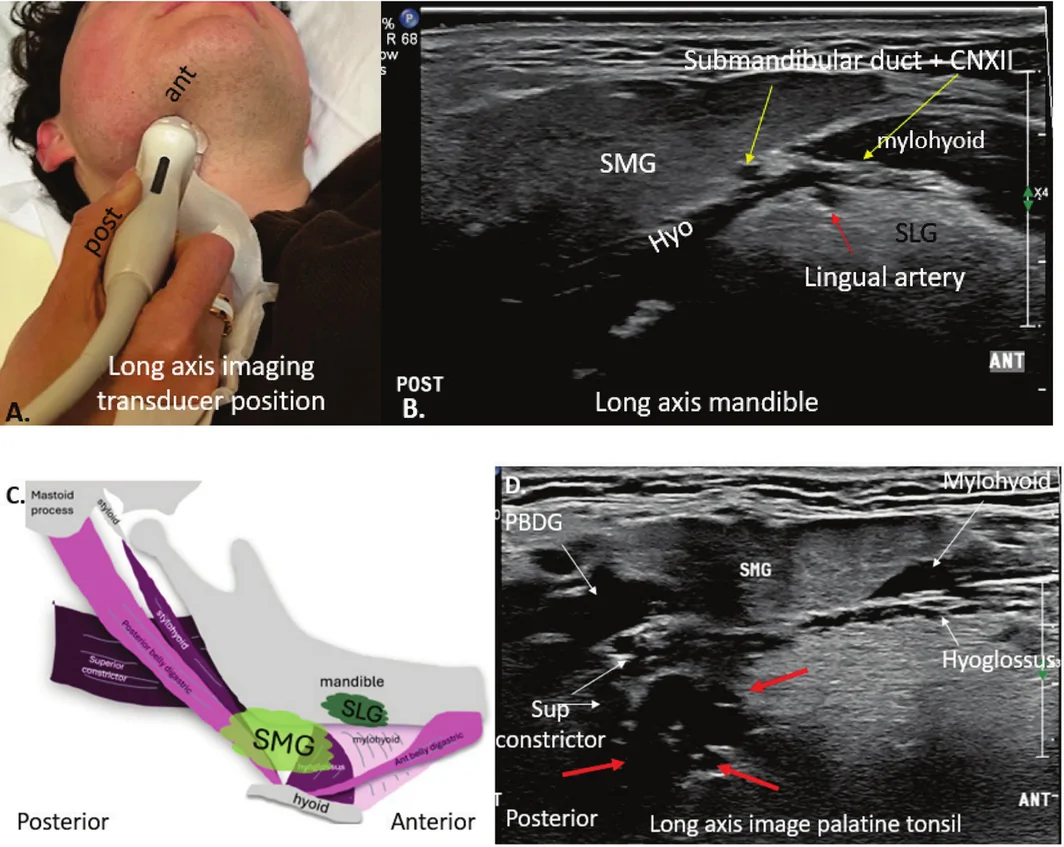
Long axis imaging of the palatine tonsil. (A) Transducer positioning for long axis trans‐cervical imaging in a sagittal oblique plane. (B) Long axis submandibular sonographic image outlining anatomy seen superficial and anterior to the palatine tonsil including the mylohyoid and hyoglossus (Hyo) muscles. (C) Figure demonstrating the muscular anatomy used as sonographic landmarks to assist identifying the palatine tonsil. (D) Long axis image of the palatine tonsil (outlined by red arrows). The submandibular gland (SMG) can be seen superficial to the tonsil, acting as an acoustic window. The mylohyoid, hyoglossus, posterior belly of digastric (PBDG) and the superior constrictor muscle is outlined (white arrows). ANT, anterior aspect; CNXII, hypoglossal nerve, cranial nerve 12; POST, posterior aspect; SLG, sublingual gland; Sup constrictor, superior constrictor muscle.

### 3.6 Short Axis Trans‐Cervical Sonographic Imaging of the Palatine Tonsil

For short axis imaging of the palatine tonsil, the transducer is rotated 90 degrees from long axis into a coronal oblique plane [36]. The SMG will still be seen superficial to the palatine tonsil. The lateral edge of the transducer is usually placed over the mandible, which is represented by posterior acoustic shadowing on the lateral aspect of the image. The oral cavity and tongue will be identified on the medial aspect of the image [4, 36]. The superior constrictor muscle and paritonsillar space may be seen lateral to the tonsil, between the tonsil and mandible; however, this can be obscured by shadowing of the mandible (Figure 6).

**FIGURE 6:**
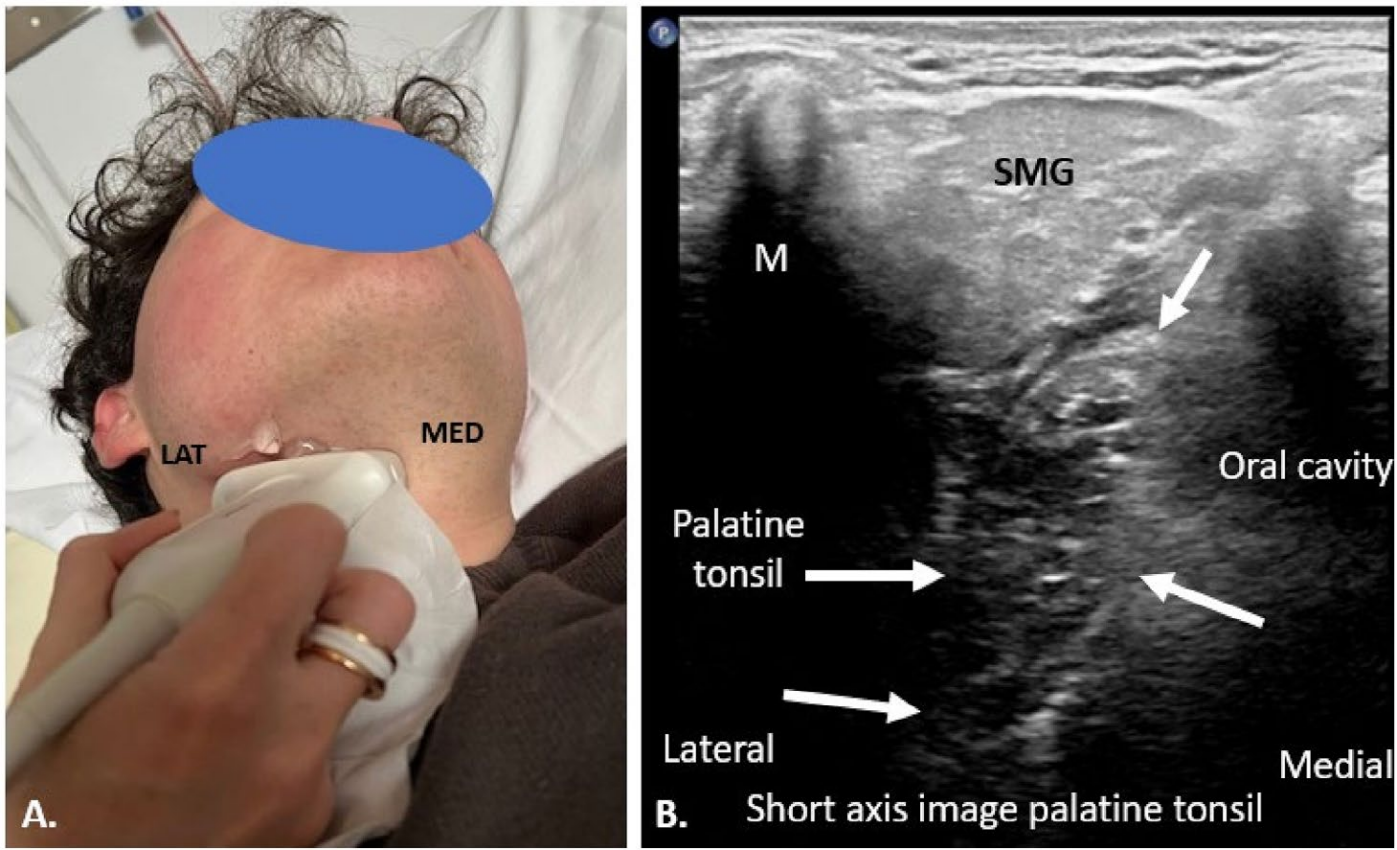
Short axis trans‐cervical sonographic imaging of the palatine tonsil. (A) Transducer positioning for trans‐cervical short axis imaging. (B) Sonographic short axis image of the right palatine tonsil (outlined by white arrows) deep to the submandibular gland (SMG). LAT, lateral aspect; M, mandible; MED, medial aspect.

It can be challenging to discriminate the tonsil with air in the medially located oral cavity; hence, identification and annotation of the relative aspects of the ultrasound image are important. The mucosal edge of the base of the tongue, medial to the tonsil, appears as an echogenic line, useful for image orientation [37]. The tongue position can be confirmed by moving it during dynamic ultrasound imaging. Additionally, the patient can be asked to swallow to aid discriminating between the oral cavity and the palatine tonsil (Video 1).

**VIDEO 1:** Long and short axis sonographic imaging of the palatine tonsils, with real time sweeps and swallowing demonstrating dynamic ultrasound. Video content can be viewed at https://onlinelibrary.wiley.com/doi/10.1002/jmrs.70139.

## 4 Pathology

### 4.1 Inflammatory Pathology of the Palatine Tonsils and Peritonsillar Tissues

Tonsillar and peritonsillar inflammation can occur both unilaterally or bilaterally [41]. Inflammatory tonsillar pathology includes uncomplicated tonsillitis, peritonsillar cellulitis, intratonsillar abscess and peritonsillar abscess, unpacked below (Figure 7).

**FIGURE 7:**
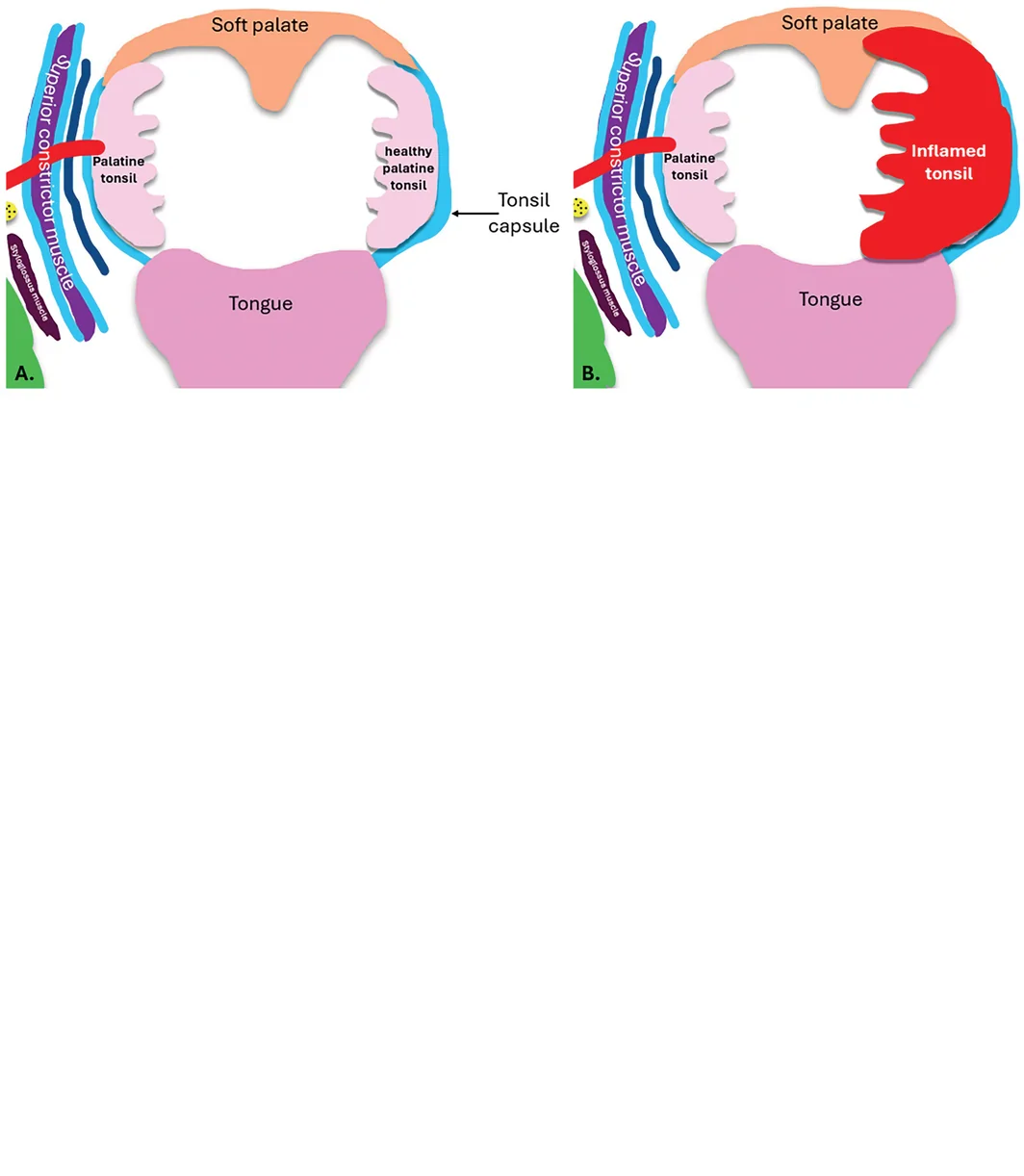
Spectrum of inflammatory tonsillar pathology. (A) Healthy palatine tonsil. (B) Uncomplicated tonsilitis; tonsils enlarged, (C) Peritonsillar cellulitis (inflamed peritonsillar tissues), (D) Intratonsillar abscesses, (E) Peritonsillar abscess and (F) Extension of abscess beyond the peritonsillar space.

#### 4.1.1 Uncomplicated Tonsillitis

Tonsillitis, typically affecting the palatine tonsils, is a common viral or bacterial condition [10]. When tonsillitis is an isolated finding, it is referred to as ‘uncomplicated tonsillitis’; this manifests as tonsillar hypertrophy, without any associated fluid collections [4]. Usually occurring in children, teenagers and young adults, the most common cause of tonsillitis is the bacterial pathogen group, A beta‐haemolytic streptococcus (9GABHS) [42]. Up to 40% of asymptomatic individuals demonstrate culture positivity to this [42]. It is usually self‐limiting, with patients recovering within 4–10 days [4].

Sonographically, in cases of uncomplicated tonsillitis, the tonsils appear enlarged (> 20 mm and larger than the adjacent SMG), hypoechoic, and typically maintain a somewhat striated appearance [39]. Imaging comparison to the contralateral side is not a fail‐safe indicator of tonsillitis, as a differential in size and appearance may not be observed in cases of bilateral tonsillitis. Often the nearby jugulodigastric (JD) lymph node, also known as the tonsillar node, becomes reactively inflamed and sonographically appears enlarged and hypoechoic [43].

Grading of paediatric tonsillitis has been proposed using imaging; based on threshold tonsil measurements, using the longest tonsil dimension [4]. Mild (2–3 cm), moderate (3–4 cm) or severe (> 4 cm) measures and grades of tonsillitis are described [4]. However, quantitative sonographic measurements of the tonsils to define adult grades of tonsillitis are currently lacking and further research is needed.

#### 4.1.2 Peritonsillar Cellulitis/Peritonsillitis

Peritonsillar cellulitis, also called peritonsillitis, involves inflammation of the tissue in the peritonsillar space: between the tonsil capsule and the superior constrictor muscle [3]. The sonographic appearance of peritonsillar cellulitis commonly presents as increased thickness and echogenicity of the tissues between the tonsil and superior constrictor muscle [4]. A striated or ‘cobblestone’ appearance of these tissues may be seen [4]. Peritonsillar cellulitis can be bilateral in 20% of cases and occur in isolation or secondary to acute tonsillitis [40]. Although it can be caused by infection, it is not associated with any discrete pus collection in this space [4].

Patients with peritonsillar cellulitis are usually managed conservatively with antibiotics [3]. It is however important to distinguish it from any abscess formation that may require evacuation/drainage to ensure healing [3]. Mis‐identifying peritonsillar cellulitis for an abscess (under‐recognition of abscess formation) can result in delayed appropriate treatment, prolonged suffering and lead to complications [3].

#### 4.1.3 Intratonsillar Abscess

Intra‐tonsillar abscess formation involves a single or multiple small abscesses forming within the tonsil [40]. There is a lack of consensus on the pathophysiology of intratonsillar abscess formation [44]. However, it is typically believed to be a sequela, and complication of acute tonsillitis [40]. It is not uncommon for multiple intratonsillar abscesses to form [4, 44]. The tonsil is clinically observed to be enlarged, without any uvula deviation seen with peritonsillar abscess [40]. However, the clinical signs and symptoms of tonsillitis, peritonsillar cellulitis and intratonsillar abscess can all overlap; hence, imaging can be helpful for this discrimination [40].

Sonographically, in cases of intratonsillar abscesses, the tonsil will appear enlarged, with increased vascularity seen on colour Doppler compared to the contralateral side [40]. Intratonsillar abscesses appear as single or multiple hypoechoic or anechoic fluid collections within the tonsillar parenchyma [40]. No internal vascularity is seen sonographically within the abscesses, although they are typically surrounded by a hypervascular rim [4, 44]. Importantly, the lateral tonsil boundary and peritonsillar space should still appear well defined on imaging, distinguishing intratonsillar and peritonsillar abscesses [45].

#### 4.1.4 Peritonsillar Abscesses

Peritonsillar abscesses involve an accumulation of pus in the peritonsillar space: between the tonsil capsule and the superior constrictor muscle [40]. Having an estimated incidence between 10 and 40 cases per 100,000 annually, they are usually unilateral, although bilateral cases are reported [43, 45, 46, 47]. There are several theories as to how a peritonsillar abscess forms [3, 43]. It is commonly understood to be the final stage of an infectious continuum, where uncomplicated tonsillitis leads to exudative tonsillitis (and possibly intratonsillar abscess), which progresses to peritonsillar cellulitis, which then leads to pus accumulation in the peritonsillar space [3, 43].

Patient presentations vary, making peritonsillar abscess a challenge to clinically diagnose [7, 40]. Typically patients present in the emergency setting with severe throat pain and trismus [32]. Trismus, also known as lockjaw, describes the limited ability to open the mouth wider than 2 cm between upper and lower teeth due to spasms of the pterygoid muscles [3]. Odynophagia (painful swallowing which may lead to drooling), ‘hot potato’ voice (thick, muffled voice), a swollen uvula which is displaced to the opposite side and superior bulging anterior pillars are also other clinical signs of peritonsillar abscess [3, 6]. Further symptoms can include bad breath, ipsilateral ear ache, fever, general malaise, headache, nausea and constipation [3]. Additionally, neck lymph nodes will appear swollen and the patient can present with their head tilted toward the side of the abscess [32, 40]. Risk factors for developing a peritonsillar abscess may include smoking and chronic periodontal disease [3].

If inadequately treated, peritonsillar abscess can result in a range of complications including airway obstruction, pus aspiration from a ruptured abscess, erosion causing carotid sheath haemorrhage, and potentially further infection spread into the parapharyngeal space [6, 7]. Hence an accurate and timely diagnosis is crucial; which is where the role of imaging is important to guide timely and appropriate patient management [3, 32].

Ultrasound imaging can be used to identify pus between the tonsil and superior constrictor muscle, the main finding to indicate a peritonsillar abscess [7, 13]. The peritonsillar abscess can appear anechoic but may contain internal echoes and can demonstrate a poorly defined border [6]. Occasionally, septations or gas bubbles may be seen within the abscess [30]. The abscess outer wall will demonstrate increased vascularity on colour Doppler, whilst the abscess centre will appear avascular with no colour fill [45]. Posterior acoustic enhancement, deep to the abscess, may be seen on B‐mode imaging [13]. Abscess contents may swirl with applied transducer compression, a helpful finding to importantly differentiate a peritonsillar abscess from an enlarged, reactive lymph node [45].

The majority of peritonsillar abscesses are located superior and posterior to the palatine tonsil [39]. They can displace the tonsil medially (toward the oral cavity) and caudally [39]. Determining an abscess size on imaging is important to guide patient management as small abscesses, measuring < 20 mm may improve with antibiotics alone [30]. The relative position and distance of the abscess to the carotid artery should also be defined to assist in determining whether the abscess is drainable [39]. Ultrasound‐guided abscess drainage can be used; transcervical imaging can guide an intra‐oral drainage [6].

Sonographic identification of any parapharyngeal spread of the abscess is important. Pharyngeal abscesses within the parapharyngeal space can spread superiorly toward the head and inferiorly toward the mediastinum [4]. A key sonographic landmark for differentiating abscess spread into the parapharyngeal abscess, or differentiation of a peritonsillar from parapharyngeal abscess, is the superior constrictor muscle; with parapharyngeal abscesses being deep to the muscle and further away from the tonsil [16].

### 4.2 Non‐Inflammatory Tonsillar Pathology

Non‐inflammatory tonsillar pathology can include tonsilloliths (tonsil stones), foreign bodies, such as fishbones within the tonsils, and solid or cystic palatine tonsilar lesions [1]. Benign solid tonsillar lesions are rarer than malignant ones [48]. Most benign tonsillar lesions are reported in a younger age group and usually detected as incidental findings [48, 49]. They can include fibromas, lipomas, lymphangiomas, papillomas (sesile or pedunculated), fibroxanthomas, chondromas, mixed tumours (pleomorphic adenoma), onocytomas, ganglionuromas, neurilemmomas, or hamartomas [48]. Although mostly asymptomatic, benign tonsillar lesions may cause a sore throat, difficulty swallowing or breathing, or patients may report feeling a lump in the back of their throat [49].

Tonsillar malignancy can occur; the vast majority of tonsillar malignant tumours are squamous cell carcinomas (SCCs), which are linked to human papillomavirus (HPV) infection or heavy alcohol or tobacco use [50]. Positron Emission Tomography/CT (PET/CT) imaging is most commonly used to confirm and stage tonsillar SCCs [41]. Lymphoma (Hodgkin's and Non‐Hodgkin's), is the second most common tonsillar malignancy, and causes tonsillar enlargement [9]. Diagnosing palatal tonsillar lymphoma is clinically challenging, as its presentation may overlap with acute tonsillitis, and peritonsillar abscess [51, 52]. Further malignant tonsillar lesions can include mucoepidermoid carcinoma, adenocarcinoma, adenoid cystic carcinoma, melanoma, sarcomas, metastatic malignancies or extramedullary plasmacytomas [48, 49]. Ultrasound imaging has the potential to assist with discrimination between tonsillar malignancy and tonsillitis, as a first line of imaging investigation in adults, however, further research to investigate the sonographic size and varied appearance of adult tonsils is required.

Benign neck cysts can be commonly encountered and misinterpreted as tonsillar pathology [53]. These can include mucous retention cysts, branchial cleft cysts and thyroglossal duct cytst [48]. Other pathology that should not be confused for tonsillar pathology include mylohyoid muscle defects and ranulas [54]. The mylohyoid muscle forms a muscular base of the oral cavity, which forms a sling, inferior to the tongue [55]. When the mylohyoid muscle has a defect, it becomes discontinuous [55, 56]. Subsequently, sublingual salivary glands as well as sialoceles, fat, and or blood vessels can herniate into submandibular spaces and present as palpable masses [55]. These herniated structures should not be sonographically mistaken for peritonsillar abscesses [56]. Likewise, a ranula, which is a mucus filled cyst that originates in the sublingual gland, and its variants, the enoral randula, and more complicated plunging ranula, should also not be confused sonographically for peritonsillar abscesses [54].

## 5 Areas for Further Research

Further research is required to investigate the reliability of sonographic imaging of the adult palatine tonsils, and peritonsillar tissues. This includes investigating the sonographic appearances, measurements and sizes of contralateral healthy adult tonsils and the peritonsillar space. Such research is required to establish an evidence‐based protocol for sonographic examinations of the adult palatine tonsils and subsequent identification of tonsillar and peritonsillar pathology. Studies to investigate the ratio of tonsillar size compared to the ipsilateral SMG, would be helpful. Also, further research and work to inform ultrasound guided peritonsillar or intratonsillar abscess drainages is required.

## 6 Conclusion

Trans‐cervical ultrasound of the palatine tonsils provides a non‐invasive imaging technique to delineate the tonsils and peritonsillar tissues and diagnose tonsillar and peritonsillar pathology. Landmarks to image the tonsils include the SMG, along with the superior constrictor, mylohyoid, hyoglossus and posterior belly of digastic muscle, and the tongue and oral cavity. The peritonsilar space is readily identified between the palatine tonsil and the superior constrictor muscle. An appreciation of the anatomy, technique and imaging appearances are essential to accurately perform and interpret an trans‐cervicval ultrasound examination of the adult palatine tonsils to appropriaptely guide patient management. This can be used to improve awareness in radiology, or through integration of point‐of‐care ultrasound in emergecny and otorhinolaryngology practice.

## Author Contributions

**M.F.:** conception, design, writing, imaging, submission. **T.R.:** editing, content input.

## Funding

The authors have nothing to report.

## Ethics Statement

The authors have nothing to report.

## Consent

Individuals that had ultrasound imaging performed on them to gain normal sonographic appearances, or who were involved in static photos or videos, all gave consent for inclusion in this publication.

## Conflicts of Interest

Tristan Redden is on the editorial board of JMRS. He, however, has not been involved in any of the processing of this manuscript in an editorial capacity.

## Data Availability

Data sharing not applicable to this article as no datasets were generated or analysed during the current study.
